# Quantitative Trait Loci Involved in Sex Determination and Body Growth in the Gilthead Sea Bream (*Sparus aurata L*.) through Targeted Genome Scan

**DOI:** 10.1371/journal.pone.0016599

**Published:** 2011-01-31

**Authors:** Dimitrios Loukovitis, Elena Sarropoulou, Costas S. Tsigenopoulos, Costas Batargias, Antonios Magoulas, Apostolos P. Apostolidis, Dimitrios Chatziplis, Georgios Kotoulas

**Affiliations:** 1 Animal Breeding and Genetics, Department of Animal Production, School of Agricultural Technology, Alexander Technological Institute of Thessaloniki, Sindos, Greece; 2 Institute of Marine Biology and Genetics, Hellenic Center for Marine Research, Heraklion, Crete, Greece; 3 Molecular Population and Quantitative Genetics, Department of Aquaculture and Fisheries, School of Agricultural Technology, Technological Educational Institute of Messolonghi, Messolonghi, Greece; 4 Laboratory of Ichthyology and Fisheries, Department of Animal Production, Faculty of Agriculture, Aristotle University of Thessaloniki, Thessaloniki, Greece; American Museum of Natural History, United States of America

## Abstract

Among vertebrates, teleost fish exhibit a considerably wide range of sex determination patterns that may be influenced by extrinsic parameters. However even for model fish species like the zebrafish *Danio rerio* the precise mechanisms involved in primary sex determination have not been studied extensively. The zebrafish, a gonochoristic species, is lacking discernible sex chromosomes and the sex of juvenile fish is difficult to determine. Sequential protandrous hermaphrodite species provide distinct determination of the gender and allow studying the sex determination process by looking at the mechanism of sex reversal. This is the first attempt to understand the genetic basis of phenotypic variation for sex determination and body weight in a sequential protandrous hermaphrodite species, the gilthead sea bream (*Sparus aurata*). This work demonstrates a fast and efficient strategy for Quantitative Trait Loci (QTL) detection in the gilthead sea bream, a non-model but target hermaphrodite fish species. Therefore a comparative mapping approach was performed to query syntenies against two other Perciformes, the European sea bass (*Dicentrarchus labrax*), a gonochoristic species and the Asian sea bass (*Lates calcarifer*) a protandrous hermaphrodite. In this manner two significant QTLs, one QTL affecting both body weight and sex and one QTL affecting sex, were detected on the same linkage group. The co-segregation of the two QTLs provides a genomic base to the observed genetic correlation between these two traits in sea bream as well as in other teleosts. The identification of QTLs linked to sex reversal and growth, will contribute significantly to a better understanding of the complex nature of sex determination in *S. aurata* where most individuals reverse to the female sex at the age of two years through development and maturation of the ovarian portion of the gonad and regression of the testicular area. [Genomic sequences reported in this manuscript have been submitted to GenBank under accession numbers HQ021443–HQ021749.]

## Introduction

Sex determination is one of the most important mechanisms in biology as well as one of the cornerstones of evolution guaranteeing sexual reproduction. Sex determination is the process by which an organism is assigned to male or female gender. Sex-linked diseases are linked to sex determination that differs significantly among species. It is to be distinguished from sex differentiation that describes the process where an undifferentiated gonad turns into an ovary or a testis. Among vertebrates teleosts present a considerably wide range of sex determination patterns, which may be influenced by environmental parameters such as temperature or group dynamics (abiotic environmentally-controlled sex determination, ESD) or is genetically determined (genetic sex determination, GSD) (for reviews see [1], [2], [3]). Nevertheless, the precise mechanisms involved in primary sex determination of fish have not been studied extensively. On the other hand, the process of sex differentiation has been the subject of several studies in fish. Interestingly, different reproduction types in fish may occur among closely related species (e.g. tilapia, *Oreochromis spp*.) or even among populations of the same species (e.g. platyfish, *Xiphophorus maculatus*) (for a review, see [4]). Furthermore, the Teleostei infraclass is the only vertebrate class covering the complete range of sexuality from gonochorism to natural hermaphroditism (either simultaneous or sequential). Up until today, model organisms in fish have been selected on the basis of several features that are important mainly for developmental studies. Thus, even in the most common model fish species like the zebrafish (*Danio rerio*), the mechanisms of sex determination remain unresolved [5]. Model fish species are ideal for studying physiological and developmental processes, but they mainly represent gonochoristic species, lacking discernible sex chromosomes and the sex of juvenile fish is difficult to determine. Sequential protandrous hermaphrodite species provide distinct determination of the gender and sex determination studies can be performed through the possibility of studying the mechanism of sex reversal. Consequently hermaphrodite fish species can become a target group to study sex determination although they are not belonging to the species for which extended genome information is available. However, due to the increasing interest in aquaculture fish species, research has been made in their physiology and in molecular biology while the need for genomic resources has been addressed and is developing as for example for the gilthead sea bream *Sparus aurata*, a sequential protandrous hermaphrodite. For commercially important gonochoristic species like the European seabass which, like the gilthead sea bream bred communally, genotypic correlations for sex have been reported [6]. Non-monogenic traits of interest like sex determination and growth in non-model species can be detected through quantitative trait loci (QTL). Quantitative genetic variation characterizes most traits in livestock, including growth-related traits and disease resistance. Interestingly, a genetic correlation between body weight and sex reversal in gilthead sea bream was shown in 1998 by pedigree based genetic parameter estimation [7]. Correlation among growth related traits and male age at maturation was reported in the rainbow trout (*Oncorhynchus mykiss*) [8]. In the Atlantic salmon (*Salmo salar*), a QTL for maturation was identified which is close to a QTL affecting body weight. QTL markers for body weight and maturation timing were also reported for the Arctic char (*Salvelinus alpinus*) [9]. In most aquaculture fish species, sex ratio influences significantly the structure of populations and determines their reproductive potential [3]. In the case of *Sparus aurata*, a sequential protandrous hermaphrodite, individuals at their early stages of sexual development have undifferentiated gonads, which mature as males at the final stage of the first reproductive cycle. From the second reproduction year on they turn to females through development and maturation of the ovarian portion of their heterosexual gonad and regression of the testicular area. Zohar et al. [10] stated that sex reversal is controlled by group dynamics and social activities. Specifically, old males have a higher potential than younger males do to experience sex reversal, and the presence of high females to male ratio inhibits the sex reversal of males. Several studies have dealt with the gilthead sea bream sex change including physiology studies [10], [11], [12] as well as gene expression studies [13], [14]; however, sex chromosomes or sex-linked genetic loci still await identification. To date molecular studies have been mainly focused on the identification of genes that establish sexual dimorphism such as the transcription factor *dmrt1* in testis differentiation and cytochrome P450 aromatase in ovarian differentiation. In fact, the major sex-determining gene characterized in teleost fish is the *DMY* gene in medaka (*Oryzias latipes*) [15]. The molecular toolbox produced for the gilthead sea bream which includes a first linkage map (LG map) based on some 200 microsatellite markers [16], EST (Expressed Sequence Tag) studies (e.g. [17], [18] as well as the construction of the first radiation hybrid map (RH map) of the species [19] has paved the way for genetics and genomic investigations of the molecular mechanism of sex reversal. Nevertheless, there is still need for functional and physiological studies while the absence of genome information makes QTL studies tedious and laboratory intensive. The existing molecular toolbox, on the other hand provides the possibility to perform comparative genomics analysis in order to delineate candidate regions for the traits of interests. Thus, information coming from other fish species, either model species or economically important species can be mapped onto the existing RH and LG map [20]. In this manner more targeted QTL scan is performed.

In the present study we performed comparative analysis via the three-spined stickleback (*Gasterosteus aculeatus*) genome, of gilthead sea bream to two species with different sexuality for which growth-related QTL were detected. The first species is the gonochoristic European sea bass *Dicentrarchus labrax* (Moronidae); here a QTL for growth was detected at linkage group 1 [21]. The second species, the Asian sea bass *Lates calcarifer* (Latidae), is like the gilthead sea bream, protandrous hermaphrodite. For the Asian sea bass a QTL for growth was detected at linkage group 2 [22], [23]. In order to select candidate regions from *Sparus aurata* genome for QTL scanning, we looked for the homologous groups for European seabass LG1 and Asian seabass LG2. In addition the present QTL scan is also linked to the results retrieved through comparative analysis to *Tetraodon nigroviridis* [20] where a candidate region for sex determination was proposed.

## Results

### Parentage analysis and population parameters

Parentage assignment of sea bream progeny (552 fish), using nine microsatellites loci, indicated that 409 individuals (74.6%) were unambiguously assigned to only one pair of parents, 47 individuals (8.5%) showed multiple parental assignment (progeny could be assigned to more than one pair of parents) and 93 individuals (16.9%) showed no assignment due to no match at one or more loci or due the absence of PCR product for these loci. Simulations performed, in order to assess the confidence of parentage assignments, showed that the proportion of correct allocation of parental pairs, using the specific set of loci, was 95%. The resulting pedigree structure from the parental assignment analysis is presented in Table 1. In summary, 10 male and 48 female brooders contributed to a structure of ten paternal half-sib families with 360 males and 49 females. It has to be noted that the percentage of females accounted to originally 3% (62 females out of 2146 offspring). The average body weight (BW) of the 409 individuals was 691.7 g. Male progeny had a mean weight of 697.8 g (132–1055 g) whereas the respective value for the female offspring was slightly lower (647.2 g, in the range 248–1085 g). There was a significant difference between male and female bodyweight (*P*<0.05). Descriptive statistics for body weight are given in Table 2. Genotyping for the genome scan confirmed to a great extend the parentage assignment using 9 microsatellites with the PAPA software as only 10 offspring out of 419 were excluded from the QTL analysis when the rest of the microsatellite markers were genotyped. Given the 5% allowance for error rate by PAPA, the 2.4% incorrect parentage assignment using 9 microsatellite markers seems to be within the margin allowed by the software.

**Table 1:** Pedigree structure, derived from parentage analysis, comprising of ten paternal half-sib families with totally 409 sea bream progeny.

Half-sib family	1	2	3	4	5	6	7	8	9	10
Male broodstock ID										
	BR_064	BR_010	BR_009	BR_090	BR_173	BR_129	BR_168	BR_094	BR_067	BR_085
**Female broodstockID (BR_)**	1,11,12,17,20,24,28,34,43,60,79,101,171 (*n* = 13)	12,24,43,71,74,76,108,110,112,117,131,138,143,145,154,158 (*n* = 16)	4,12,24,27,61,79,104,122,125,142,171(*n* = 11)	15,24,28,79,108,109,143,145,162 (*n* = 9)	2,12,21,51,59,79,108,109,117,125,138,142,162,171(*n* = 14)	2,12,20,45,51,122,144,171 (*n* = 8)	2,12,24,51,60,66,71,75,76,79,93,104,113,122,138,142,145,171 (*n* = 18)	79,109,145 (*n* = 3)	24,27,28,43,51,75,76,109,122,144,162(*n* = 11)	37,79,109 (*n* = 3)
**Number of offspring**	115	65	45	35	40	19	61	9	13	7
										*Total* = 409

BR_ means brooder.

**Table 2:** Mean body weight (g) and standard deviation (S.D.) of *S*. *aurata* male, female and all offspring.

	Mean body weight (g)	S.D.	Minimum	Maximum
All offspring (*n* = 409)	691.706	±150.733	132	1085
Males (*n* = 360)	697.786	±144.515	132	1055
Females (*n* = 49)	647.163	±186.819	248	1085

### Comparative analysis

**Figure 1:**
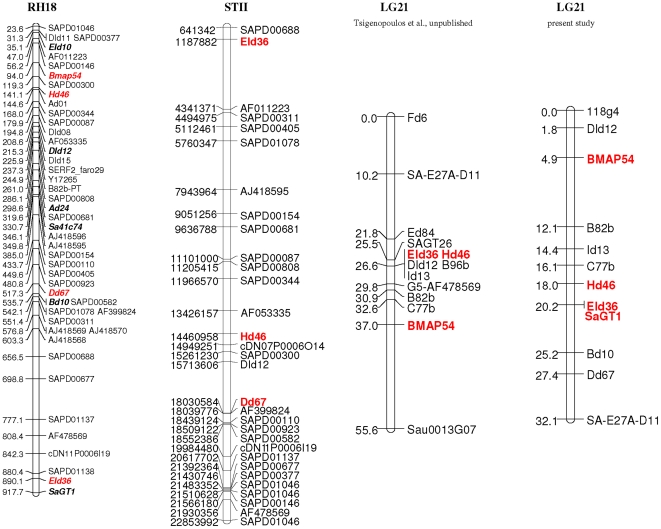
Radiation hybrid group 18 in comparison to linkage group 21. *Sparus aurata* radiation hybrid group 18 (RH18, [20]), genetic linkage group 21 (LG21) and homologues found in three-spined stickleback (*Gasterosteus aculeatus*) chromosome STII. Highlighted in bold red are the candidate microsatellite markers linked to growth and sex. Highlighted in bold are the genotyped markers for the QTL analysis.

**Figure 2:**
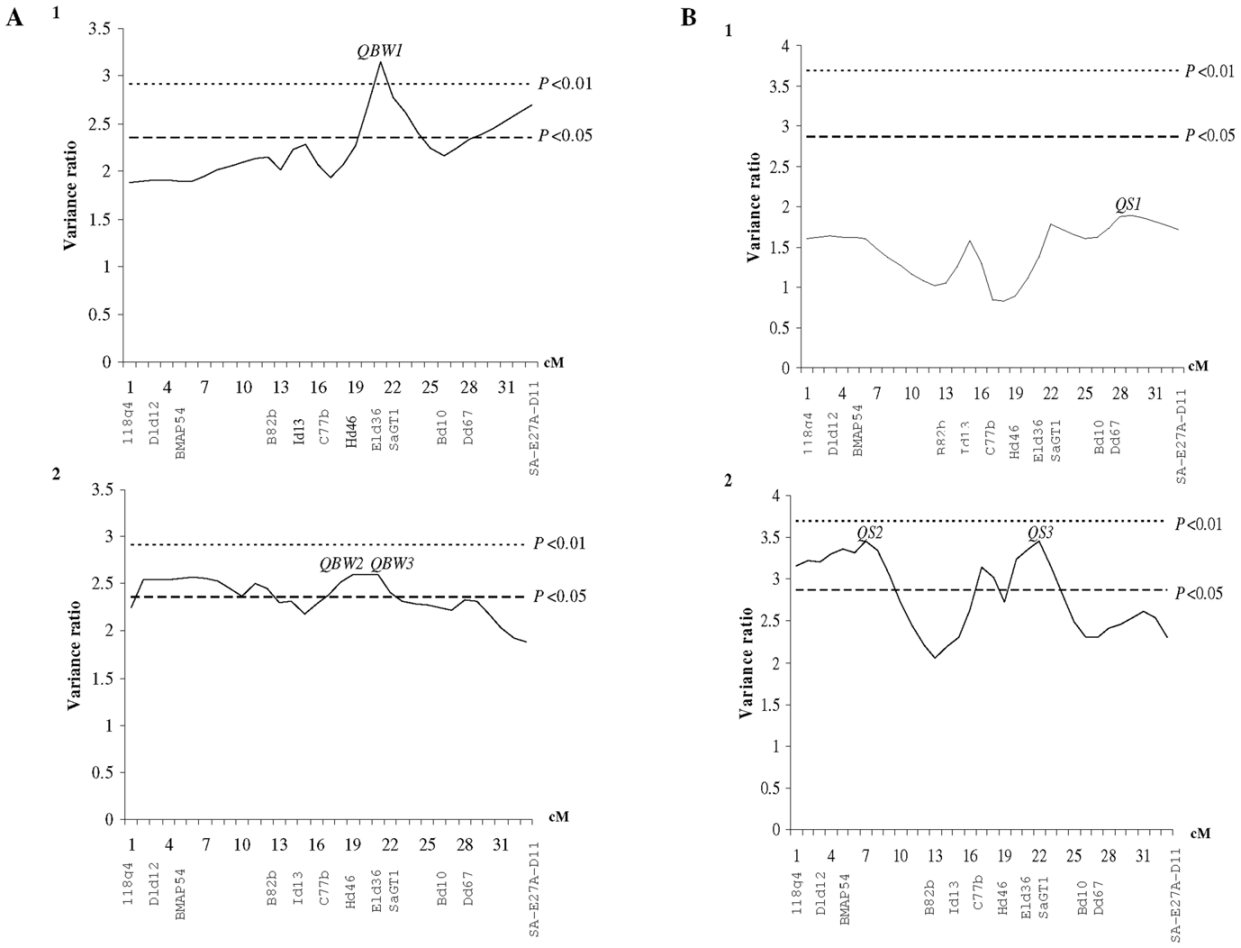
Quantitative trait loci for sex and growth. A. Mapping of QTL for body weight in *Sparus aurata* LG21: 1. One-QTL model for body weight; 2. Two-QTL model for body weight. B. Mapping of QTL for sex in *Sparus aurata* LG21: 1. One-QTL model for sex; 2. Two-QTL model for sex.

Growth related QTL detected in the European seabass linkage group 1 (DL-LG1) [21] and in the Asian seabass linkage group 2 (LC-LG2) [22], [23] were mapped to stickleback chromosome V (STV) and STII which correspond to sea bream RH groups (SA-RH) 11 and 18 respectively. Moreover, according to comparative mapping results, between linkage and RH maps in sea bream, SA-RH11 corresponds to SA-LG2 and SA-RH18 corresponds to SA-LG 21 (Fig. 1) from the second generation linkage map of *S. aurata* [20]. Therefore, fourteen microsatellite markers from SA-RH18–SA-LG21 groups (Figs. 1 and 2) and ten microsatellite markers from SA-RH11–SA-LG2 groups were selected for genotyping brooders and their offspring.

### Linkage mapping of microsatellite markers

Twelve out of fourteen genotyped microsatellite markers from *S*. *aurata* RH 18-LG21 (Fig. 1) were polymorphic, the remaining two microsatellite markers (Ad24 and Sa41c74) were monomorphic and subsequently removed from the quantitative analysis. The number of alleles for these markers ranged from 3 to 22 (mean  = 9). According to the results of linkage analysis the 12 microsatellites were mapped, as expected, onto the same linkage group (SA-LG21, Fig. 1) with a total length of 32.1 cM. However, exact collinearity of markers between SA-LG21 and SA-RH18 could not be established. The 12 markers are relatively close to each other, showing a mean interval of 2.92 cM.

For the SA-RH11-LG2 group, all 10 genotyped microsatellites were polymorphic (2–18 alleles, mean  = 7.1). Linkage analysis confirmed linkage of the above ten markers onto the same linkage group (SA-LG2), with a total length of 64 cM and mean marker interval of 7.09 cM. In addition analysis showed no difference in the loci order of SA-LG21 and SA-LG2 groups in spite of the assumption of different recombination rate between sexes.

### Analysis of BAC sequences

For eight microsatellite markers, namely Hd46, SAPD00344, Did12, AJ418596, AJ418568, AF478569, SAPD00688 and SAPD00677 located in the region of interest, BAC clones were sequenced, thus genomic information around each marker is available for SNP (Single Nucleotide Polymorphism) detection (Fig. S1). BAC clones sequences are mapped onto stickleback chromosome II and in total 1,325,954 base pairs (bp) are assembled into 307 contigs (∼12 contigs for each BAC). For parts of the two main regions, STII 14 Mb and STII 15 Mb, which have been found to be genetically linked, genome information was obtained based on sequences of 8 (71 contigs) and 5 (26 contigs) overlapping BAC clones (Fig. S2 and S3). The remaining sequences of the other 13 BAC clones were mapped independently over ST II with one BAC clone, breambac-141b24, being re-located on ST XVI instead of ST II.

The two regions, in which the two markers Hd46 and Did 12 were found, when mapped on the stickleback, span a genomic region of 1.58 Mb. Considering the genome size difference between stickleback and sea bream this has to be multiplied by a factor of 1.7–2 to estimate the equivalent part of sea bream's genome, which then corresponds to 2.686 Mb, under the hypothesis that there is no chromosome rearrangements in this region. This is useful information, considering that there is no recombination detected in LG21 between Hd46 and Did12 in a panel of 50 individuals (Fig. 1; [16]). Nevertheless, in the actual QTL pedigree, recombination has been detected in this region, showing that this may be a population specific parameter. Such data offer a measure of recombination rate, a parameter that in many cases is affected in a sex specific way and may constitute an indication of sex-related effects. For two microsatellite markers, namely Did12 and breambac-118g4, which were independently genotyped, linkage analysis revealed that they are separated by 1.8 cM from each other (Fig. 1). Sequence analysis showed that they are describing the same locus (Fig. S4).

### QTL detection

QTL analysis, using half-sib interval mapping [24], revealed a significant QTL controlling body weight (*QBW1*) at position 20 cM of LG21 (Fig. 2A1). *QBW1* is located between Hd46, Eld36 and SaGT1 microsatellite markers and was found to be significant both at 5% and 1% levels of significance and a chromosome-wide scale (Table 3). Moreover, when a two QTL model was fitted, two peaks (*QBW2* and *QBW3*) of the test statistic appeared on LG21 (Fig. 2A2), both significant at the 5% level (Table 3). However, the comparison between the two models (Table 3) suggested that the one-QTL model was of better fit (showing a higher variance ratio). On the other hand, fitting one- and two-QTL models on LG2 showed no significant association between any of the markers and body weight. Test statistics, significance thresholds, mean size of effect and position of detected QTL on LG21 for one and two QTL models are presented in Table 3.

**Table 3:** QTL variance ratios (*F*-statistic) and significance critical values (determined by permutations, Churchill and Doerge, 1994) at 1% and 5% levels for body weight and sex obtained in one and two QTL models for *S*. *aurata* linkage group 21 (LG21).

One QTL model									
Trait	QTL name	Nearest marker(s)	Variance ratio	Critical value			Position (cM)	Mean effect	Standard error
				*P*<0.05		*P*<0.01			
Body Weight	*QBW1*	*Hd46,Eld36,SaGT1*	**3.42** **	2.21	2.99		20	30.78 (g)	12.7
Sex	*QS1*	*Dd67*	1.90	2.86	3.69		28	0.05	0.03

Two QTL model												
Trait	QTL name		Nearest marker(s)		Variance ratio 2		Critical value		Position		Mean effect	Standard error
	QTLA	QTLB	QTLA	QTLB	2 QTL vs. 0 QTL	2 QTL vs. 1 QTL	*P*<0.05	P<0.01	(cM) QTLA	QTLB		
Body Weight	*QBW2*	*QBW3*	*Hd46*	*Eld36 SaGT1*	**2.57** *	1.89	2.21	2.99	18	19	26.82 (g)	14.1
Sex	*QS2*	*QS3*	*BMAP54*	*Hd46 Eld36 SaGT1*	**3.45** *	**4.33** **	2.86	3.69	6	20	0.14	0.02

* Significant in *P*<0.05 level at a chromosome-wide scale.

** Significant in *P*<0.05 and *P*<0.01 levels at a chromosome-wide scale.

Regarding QTL detection for sex determination, under the assumption of one QTL model, the analysis did not reveal any significant QTL on LG21 (Fig. 2B1, Table 3). However, when a two-QTL model was fitted, two peaks (*QS2* and *QS3*) of the test statistic appeared on LG21 (Fig. 2B2) both significant at the 5% significant level at a chromosome-wide scale (Table 3). The two QTLs are located at position 20 cM of LG21 (Fig. 2), as it was the case for *QBW1* affecting body weight and at 6 cM which is 1.1 cM far from marker BMAP54 (Fig. 2). The test statistic for the two-QTL model was quite high (Table 3), despite the low percentage of females in the offspring panel (12%). Finally, QTL analysis in showed no significant QTL associated to sex determination in LG2, using both QTL models.

## Discussion

### Parentage analysis and population parameters

Most methods used for assigning parents to offspring do not provide a 100% confidence of correct assignment, but usually allow between a 1% and 5% level of error, as was the case in the present study. However, such pedigree mistakes result in a small loss of QTL detection power [25] and can be improved when individuals are genotyped with more markers for linkage analysis and QTL mapping purposes. Since the gilthead sea bream is a sequential mass spawner the contribution of 10 male and 48 female brooders to the population structure in the present study was expected [26]. Offsprings were collected at the age of 2 and sexing was performed by stripping. The percentage of females amounted to 3% (62 females out of 2146 offspring). The small presence of females in the progeny was expected, as old males have a much higher potential to go through sex reversal than younger males do, thus at two years of age only a few male individuals have turned into to the female sex. The exact time point of fish changing sex varies. The percentage of females during the second year has been reported to vary from 15% to 80% [10], [27], [28], and in the third year this percentage may increase to 63% [28]. However another study showed that gonadal development is at the end of the second and beginning of the third reproduction cycle [13], emphasizing the population specific behavior of sex reversal. For analysis purposes the structure of ten paternal half-sib families with 360 males (∼88%) and 49 (∼12%) females were chosen.

### Comparative mapping approach in QTL studies

The rapid developments in genomics of fish paved the way to new and valuable research in comparative genetics and genomics. With the accumulation of genomic information in model species, the genetic and genomic characterization for non-model, but economically important species such as the gilthead sea bream, is now feasible. Only recently Massault et al. [29] identified two QTLs for body weight; DL-LG4 and DL-LG6. Corresponding RH groups here are RH 2 and 12, respectively. In seabream recently Massault et al. [29] described a QTL for body length on RH1/LG6 [30]. In addition, the comparison of low coverage gene maps of aquacultured fish species against the fully sequenced fish species enhances the efficiency of candidate genes identification projected for QTL scans for traits of interest. Specifically, the previous study from Sarropoulou et al. [20] demonstrated a well-conserved gene order between SA-RH18 and chromosome 5 of *Tetraodon nigroviridis*. In this particularly well-conserved region of *Tetraodon* chromosome 5 the genes for gonadal P450 aromatase (or cyp19a1a) and brain aromatase (or cyp19a1b, neural aromatase, P450aromB) were located [20]; the former is a neural marker of estrogen effect known to be involved in sex differentiation [31], [32], [33], the latter catalyzes the key step in estrogen biosynthesis [34], [35] and is also a neural marker of estrogen effect in teleosts. In addition, SA-RH18 and *Tetraodon* chromosome 5 correspond to LG1 of tilapia [20], in which a candidate sex-determining region was mapped [36], [37]. Our results, regarding the detection of two sex-linked QTL in SA- LG21 (Fig. 2, Table 3), testify the great power of comparative genomics for targeted QTL scanning.

The insufficient genomic information for direct comparison between sea bream the European sea bass and the Asian sea bass, for which growth-related QTL were detected [21], [22], was bridged by comparative mapping through the fully sequenced genome of stickleback. The highest percentages of significant hits by BLAT analysis for *S*. *aurata* were obtained by comparing it to the three-spined stickleback (58%), followed by medaka (32%) and *Tetraodon* (30%) [38]. Thus, stickleback was used as stepping stone that allowed passing from the European and the Asian sea bass linkage groups of interest (DL-LG1 and LC-LG2, respectively) to their gilthead sea bream homologous RH groups (SA-RH11 and SA-RH18, correspondingly). Looking at the biology of both species, the European sea bass is a gonochoristic species with a spawning period between December and March in the Mediterranean and up to June in the Atlantic, whereas the Asian sea bass belongs to the protandrous hermaphrodites [39] like *S*. *aurata*, and is a diadromous fish which spawns in estuaries and inhabits rivers. QTL affecting growth-related traits in sea bream was detected in SA-RH18 (LG21; Fig. 2 and Table 3) corresponding to LC-LG2 of Asian seabass. However, no statistical significance for QTL affecting growth-related traits was found in SA-RH11 the corresponding LG of European sea bass (DL-LG1) for which linkage to growth has been shown by Chatziplis et al. [21]. We suggest that targeted QTL scans through comparative genomics can be even more powerful when also taking into account the physiological characters of the examined species.

### QTL analysis for body weight and sex determination

Optimal designs for QTL detection depend on the specific characteristics of a species and, therefore, appropriate designs appropriate for terrestrial livestock species may be not transferable to aquaculture species. A major complication in mass-spawning fish species is that, in general, the effective number of parents is much lower than their total number. Consequently, the contribution of different individuals may extremely vary and in some cases brooders do not even contribute at all [26]. This mode of reproductive behavior is also reflected in our data where, from a panel of 16 males and 109 females brooders, 10 males and 48 females have finally participated to the creation of ten paternal half-sib families with family size ranging from 7 to 115 progeny. On the other hand, simulation studies underline the importance of family structure in experimental design and its major effect on the power to detect QTL [25]. More specifically, when a QTL, explaining 15.5% of the phenotypic variation, is segregating, it was shown that analyzing half-sib family designs with a regression method, a power of 80% for QTL detection can be obtained by using five families with 100 offspring each when a QTL [25], [40]. As a general rule it is suggested to analyze fewer larger families than more families with fewer offspring. In practice, such large and equally sized families are difficult to obtain in mass-spawning species like *S*. *aurata*, and it is more likely to have a variety of family sizes due to the unequal contribution of brooders. In the last case, more individuals should be genotyped to ensure lots of offspring per family. However, given the family size and structure in the present study, sufficient power for QTL detection can be assured. In addition the domestication process was in its early phase and since no systematic selective breeding has been operated the populations possess high levels of genetic variation that makes them ideal for QTL detection through full- and/or half-sib families.

Seabream is considered a sequential protandrous hermaphrodite species. Karyotype analysis has not revealed the existence of sex chromosomes [41]. Therefore, in the QTL analysis of sex determination (positions 6 and 20 cM on LG21; Fig. 2B2), the sex of the fish was treated as polygenic binary trait scored as one (male) or two (female). The same regression analysis was used for body weight [24], since simulation studies showed that regression methods for QTL mapping are also applicable to binary traits even in unbalanced half-sib family designs [42], [43], [44]. When applying the linear regression method in an unbalanced multi-family half-sib design of 500 progeny and for a 15% incidence of the binary trait, Kadarmideen et al. [42] suggested that a power of 88.4% for QTL detection of binary traits can be obtained at *P*<0.05 level of significance.

Body weight was significantly associated with a QTL (*QBW1*; Fig. 2A1) in position 20 cM of LG21. *QBW1* seems to be tightly linked to body weight, as it was found significant at both 5% and 1% levels of significance at a chromosome-wide scale (Table 3). Furthermore, comparison between the one- and two-QTL models shows that the existence of a single QTL is more likely to be affecting the body weight in the linkage group 21 of *S*. *aurata*. The most likely position for *QBW1* is between microsatellite markers Hd46, Eld36 and SaGT1 (Figs. 1 and 2A1). BAC sequence data provided here which include mapped microsatellite markers, confirm mapping results, will enable fine mapping of the detected QTL as well as further comparative studies.

### Sex Determination (SD) region of gilthead sea bream

Elucidation of the full spectrum of processes that lead to sex change in gilthead sea bream requires detailed data derived from physiological, molecular, cytogenetic, genomic and genetic surveys. Up to date, the mechanism of *S*. *aurata* sex reversal is thought to be controlled by social activities and group dynamics [10], [11]. Changes in these environmental factors are the signals received by the brain and finally lead to differential expression of specific genes (*cyp19a1a* [14]) during sex change. Our study provides strong evidence for the presence of genetic control in sex reversal of this teleost fish. The two sex-linked loci detected in SA-LG21 group (*QS2* and *QS3*; Fig. 2) were found significant at the 5% level of significance whilst their variance ratio was also very close to the 1% threshold value (Table 3), despite the imbalanced design comprising 12% of females in the offspring panel. Another noticeable result herein is that *QS3* is located at the same position of SA-LG21 (20 cM) as the *QBW1* affecting body weight (Table 3). This pleiotropy between QTL could explain partially the genetic correlation between body weight and sex reversal in sea bream, suggested also in Batargias [7] by pedigree-based genetic data analysis (i.e. genetic parameter estimation), and it further supports our hypothesis of genetic control in sex determination apart from environmental factors. In addition the finding of the QTL for weight and the QTL for sex at the same genomic position is in line with newer synthesis about sex determination in fish.

In the case of sea bream, it has been shown that the contribution of males to next generation follows a concave distribution [45], [46], and after a certain size it is negatively correlated with weight, showing that there is an evolutionary trade-off between male size and sex reversal. The nature of this interaction is not known, but having defined one possible genomic region where the phenomenon is located, together with the power of new generation sequencing keeps promise for reaching deep insights in the genetics and the evolutionary drivers of sex determination in fish. Our findings combined with results from previous studies may not be limited to this species but could potentially yield further insight into the general phenomenon of hermaphroditism, as this reproduction pattern is observed in a significant number of fish and especially in sparids.

In conclusion, we demonstrated a fast and efficient strategy for QTL detection through comparative genomics and linkage analysis. We identified in this way two significant QTLs, one for body weight and one QTL affecting sex determination, both located in linkage group 21. Our results were verified in ten paternal half-sib families comprising of totally 409 progeny. To our best knowledge, this is the first report of QTL detection associated with sex and body weight in *Sparus aurata*. In addition, we provide BAC sequence information for SNP detection in order to enable fine mapping of QTL as well as comparative studies. Having defined one possible genomic region where linkage to sex determination has been shown together with the power of next generation we expect to obtain access to the molecular basis of nature of sex reversal as well as to the relation of sex reversal and growth.

## Materials and Methods

No ethic statement is required for this study. Fish handling was carried out according to the European Union Directive (86/609EEC) for the protection of animals used for experimental and other scientific purposes [47] and the “Guidelines for the treatment of animals in behavioural research and teaching” [48], [49].

### Source of fish and measurements of phenotypic traits

A commercial broodstock of 125 brooders was selected based on prior records of good performance indicating high genetic value. This broodstock consisted of 109 females and 16 males maintained under natural photoperiod. The brooders were allowed to naturally mass spawn in order to obtain relatively lots of families with large family sizes for subsequent genetic parameter estimation and QTL analysis.

Natural mass spawning took place in April 2002. The collected eggs originated from a single spawning day. Approximately 1.6 million larvae were collected from the egg incubators and placed into two larval rearing tanks. The larvae stayed for 55 days in tanks, where they were sequentially fed with algae, rotifers and *Artemia*, and commercial feed. At the age of 55 days post-hatching, each of the larval tanks was transferred to a concrete raceway tank. Only fish from the first concrete raceway tank were used in the experiment while the second was held as a back-up population. Since the fish were originating from the same batch, were growing under identical environmental conditions (same tanks) in all rearing and growing stages of the same time period and were fed the same feed in all periods, there were no fixed effects to take into account. Consequently, there were not any fixed effects present in the experiment. The feeding regimes of differently sized commercial pellets were followed thereafter, including the on-growing period, according to age and weight.

Rearing procedure followed the company's common practice with the exception of avoiding the grading in order to preserve the whole spectrum of variance of the population. In September 2002, approximately 70,000 fish with an average weight of 7.21 g, were transferred into a circular tank appropriate for their size and mean weight. In January 2003, approximately 12,000 of the fish above, weighting 31.5 g (average), were transferred to one of the company's cage sites and were again placed in a small cage appropriate for their size and mean weight.

In April 2004, 2527 fish were gradually anesthetized with 3-phenoxy-ethanol and each fish was PIT-tagged and fin-clipped, with the fin clip preserved in absolute ethanol for further DNA genotyping. In December 2004, the fish were sampled at the cage site. During this procedure 2146 fish with mean weight of 702 g were gradually anesthetized with 3-phenoxy-ethanol, weighed to the nearest of 1 g, photographed with a Nikon digital camera and sexed by stripping. Sexing by stripping two year old sea bream means that the individuals are classified into three categories: the spermiating males (2029 individuals), indicated by the presence of sperm, the females (62 individuals), indicated by the presence of eggs, and the unidentified (54 individuals), indicated by the absence of both sperm and eggs which were probably immature or mature females, but not in ovulating condition at the time of stripping.

### Mapping panel and parentage analysis

Genomic DNA was isolated from fin clips of 125 brooders and 552 offspring from the initial offspring panel (*n* = 2146), by standard proteinase*-K* digestion, following the salting out procedure as described in Miller et al. [50]. The selected offspring consisted of 490, randomly selected, male individuals and all the female fish (*n* = 62, as described above). The quality and quantity of DNA was checked using a NanoDrop 1000 Spectophotometer (Thermo Scientific, Wilmington, DE, USA). The DNA concentration of each fish was adjusted to 20 ng/µl through dilution with distilled water and arrayed into 96-well PCR plates.

For parentage analysis, parents and offspring were genotyped with a multiplex of nine microsatellite markers, from which six (CL2416Contig1, CL120Contig1, cDN14P0004O13.F.ab1, CL2598Contig1, CL2595Contig1, cDN08P0004J06.F) were newly developed and the other three (Dd67, Hd46, Eld36) were from RH 18 (Fig. 1). The selection of the loci set was based on their polymorphism and unambiguously sized PCR product. All multiplex PCRs were performed in 10 µl volumes containing 1 unit of *Taq* polymerase (GenAxxon, Biberach, Germany), 1× *Taq* buffer S, 0.25 mM dNTPs mix, 2.5 mM MgCl_2_, 0.35 µM of each forward and reverse primer, and approximately 20 ng of template DNA. Cycling conditions for the multiplexes amplification consisted of an initial 95°C denaturation step for 3 min followed by 35 cycles of 30 s at 94°C, 90 s at 57°C, and 60 s at 72°C, with a final extension at 72°C for 10 min. Fluorescently labeled PCR products were separated on an ABI PRISM 3700 DNA Analyzer (Applied Biosystems, Inc. [ABI], Carlsbad, CA, USA), using 5′-labeled reverse primers and the GeneScanTM-500 LIZ Size Standard (ABI) as internal size standard. Alleles were sized and individuals genotyped using the software STRand 2.3.79 (http://www.vgl.ucdavis.edu/informatics/STRand).

Genotype data of brooders and offspring were analyzed using the PAPA 2.0 software [51] for parentage assignment. PAPA calculates the likelihood of each potential parental pair and if one pair has a likelihood greater than that of all the other pairs then the offspring is allocated to this pair. The allocation analysis was run without allowing for mismatching genotypes and simulations were carried out with several iterations and with the same parameter values as those used in the allocation procedure (same set of parental genotypes and same number of parents and offspring), in order to assess the correctness of allocations for parental pairs.

### Comparative mapping and selection of QTL scan groups

All sequences used in the present study were *in silico* mapped to the stickleback genome sequence using BLAT algorithm. BLAT searches were performed using q = dnax and t = dnax with a score above 80 and an alignment length above 50 bp, as is recommended for mapping ESTs to the genome across species [52]. Genome sequence data for stickleback were retrieved from the draft assembly produced by the Broad Institute at MIT and Harvard in February 2006.

### Linkage analysis

Linkage analysis and ordering of the linkage groups were performed using the CRI-MAP 3.0 software [53]. Using the ‘prepare’ option of the software, we also checked for non-Mendelian inheritance. Marker assignment to linkage groups was performed by pairwise analysis, where the marker order with the highest likelihood was selected from all the other possible orders. Linkage groups were built assuming an equal recombination rate between sexes; however, the loci order was also checked (using the ‘Flips’ option of CRI-MAP), assuming different recombination rates between sexes. The linkage distances for sex-average and sex-specific linkage groups was estimated assuming the Kosambi mapping function [54].

### BAC sequencing

Twenty-six candidate BAC clones located through *in silico* comparative mapping to stickleback chromosome II (STII, see Fig. 1) were shotgun-sequenced on a Genome Sequencer FLX 454 Sequencer using a Titanium Library Preparation kit (Roche, Basel, Switzerland). The resulting contigs were manually verified and submitted to GenBank under accession numbers HQ021443–HQ021749. Stickleback STII corresponds to *S. aurata* RH group 18 [38]. All molecular markers of RH 18 were used to locate their sequence on the sequences resulting from the 26 BAC clones. Markers physically located on one contig or within one BAC were compared to results from the linkage analysis.

### QTL mapping

Given the pedigree structure from the results of the parentage analysis results the QTL detection method was based on half-sib interval mapping analysis as described by Knott et al. [24]. For a given position of one Kosambi centimorgan (cM) interval between the marker genotypes with sex-averaged marker distances, the probability of one offspring inheriting one of its parent's alleles at that position is calculated conditional upon its marker genotype. For any given position these conditional probabilities are the independent variable on which the phenotypic score is regressed. The estimate of the position of the putative QTL is determined by the highest variance ratio and the size of each effect is estimated in that position by the regression coefficient of the analysis [24]. Since the conditional probabilities are available, various genetic and environmental models can be fitted, e.g. effects of environmental factors (fixed effects) or covariates, interactions of QTL with fixed effects etc. No fixed effects were observed in this study; however, it was possible to fit both one and two linked QTL models using sex as random effect when body weight trait was analyzed. The software GridQTL 1.3.2 (see http://www.gridqtl.org.uk) was used to perform the analysis as described by [55]. The 1% and 5% significance threshold values were determined at a chromosome-wide level, for each trait separately, using permutation techniques [56]. The phenotypic data were permutated and analyzed 1000 times and the highest variance ratio was stored after each permutation and analysis. The 10^th^ and 50^th^ highest values after sorting the 1000 variance ratios were the 1% and 5% significance thresholds for each trait, respectively.

## Supporting Information

Figure S1 Assignments of BAC clones to molecular markers mapped onto *Sparus aurata* RH18 group.
(PDF)

Figure S2 Alignment of candidate BAC clone sequences isolated around the candidate molecular marker Hd46 (breambac-141g16) for QTL affecting growth-related traits as well as sex onto the genome of three-spined stickleback (*Gasterosteus aculeatus*). Numbers on the left columns indicate nucleotide positions along the stickleback chromosome II.
(PDF)

Figure S3 Alignment of candidate BAC clone sequences isolated around the candidate region for QTL (Did12, breambac118g4) affecting growth-related traits as well as sex reversal onto the genome of Stickleback. Numbers in the left columns indicate nucleotide positions along the stickleback chromosome II.
(PDF)

Figure S4 Alignment of marker sequence Did12 and BAC sequences of *Sparus aurata* breambac-118g4. In gray are shown the corresponding primer sequences mapped 1.8 cM away from each other according to linkage mapping analysis (Fig. 1).
(PDF)
