# Supplementary material for: Quantitative Trait Loci Involved in Sex Determination and Body Growth in the Gilthead Sea Bream (Sparus aurata L.) through Targeted Genome Scan
Source: PLoS One. 2011 Jan 31;6(1):e16599. doi: 10.1371/journal.pone.0016599 (PMC3031595; doi:10.1371/journal.pone.0016599)
Supplement: Figure S1 — Assignments of BAC clones to molecular markers mapped onto Sparus aurata RH18 group. (PDF) [file pone.0016599.s001.pdf]

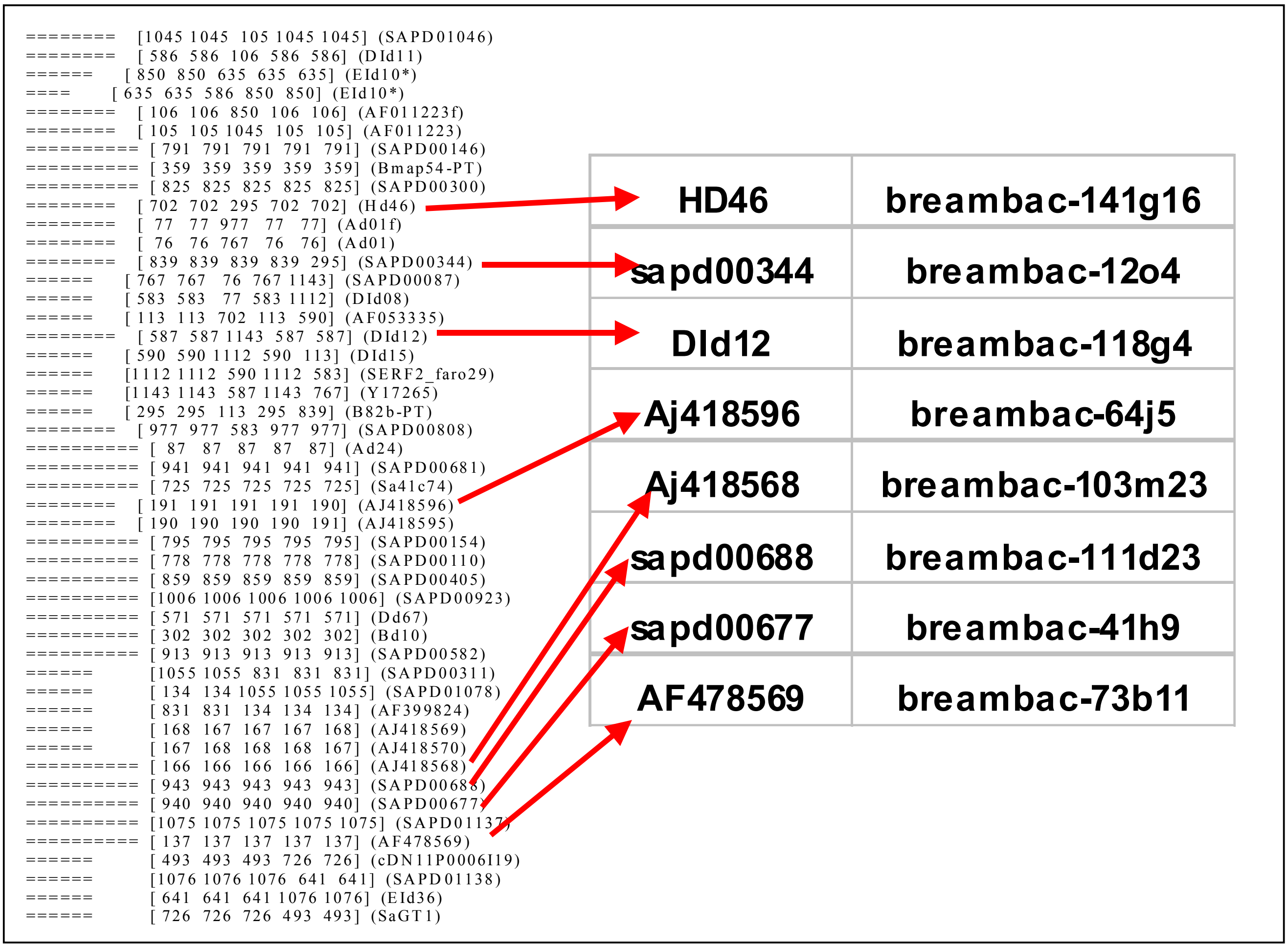

Supplemental file 1

Assignments of BAC clones to molecular marker mapped onto RH 18.
