# Supplementary material for: Quantitative Trait Loci Involved in Sex Determination and Body Growth in the Gilthead Sea Bream (Sparus aurata L.) through Targeted Genome Scan
Source: PLoS One. 2011 Jan 31;6(1):e16599. doi: 10.1371/journal.pone.0016599 (PMC3031595; doi:10.1371/journal.pone.0016599)
Supplement: Figure S2 — Alignment of candidate BAC clone sequences isolated around the candidate molecular marker Hd46 (breambac-141g16) for QTL affecting growth-related traits as well as sex onto the genome of three-spined stickleback (Gasterosteus aculeatus). Numbers on the left columns indicate nucleotide positions along the stickleback chromosome II. (PDF) [file pone.0016599.s002.pdf]

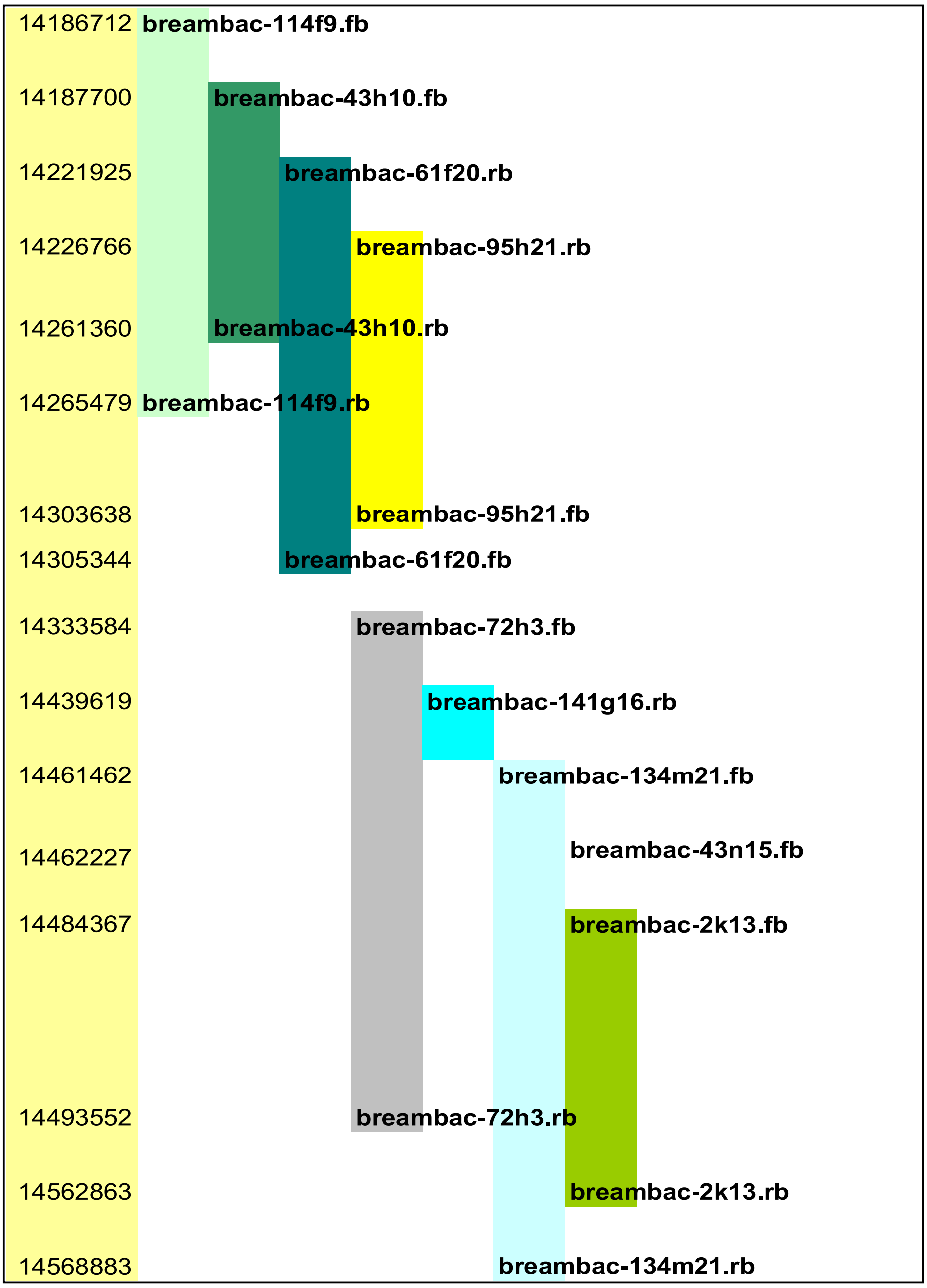

Supplemental file 2
Alignement of candidate BAC clones isolated around the candidate molecular maker Hd46 (BreamBac-141g16) for QTL affecting growth-related traits as well as sex reversal onto the genome of Stickleback.
