# Supplementary material for: Quantitative Trait Loci Involved in Sex Determination and Body Growth in the Gilthead Sea Bream (Sparus aurata L.) through Targeted Genome Scan
Source: PLoS One. 2011 Jan 31;6(1):e16599. doi: 10.1371/journal.pone.0016599 (PMC3031595; doi:10.1371/journal.pone.0016599)
Supplement: Figure S3 — Alignment of candidate BAC clone sequences isolated around the candidate region for QTL (Did12, breambac118g4) affecting growth-related traits as well as sex reversal onto the genome of Stickleback. Numbers in the left columns indicate nucleotide positions along the stickleback chromosome II. (PDF) [file pone.0016599.s003.pdf]

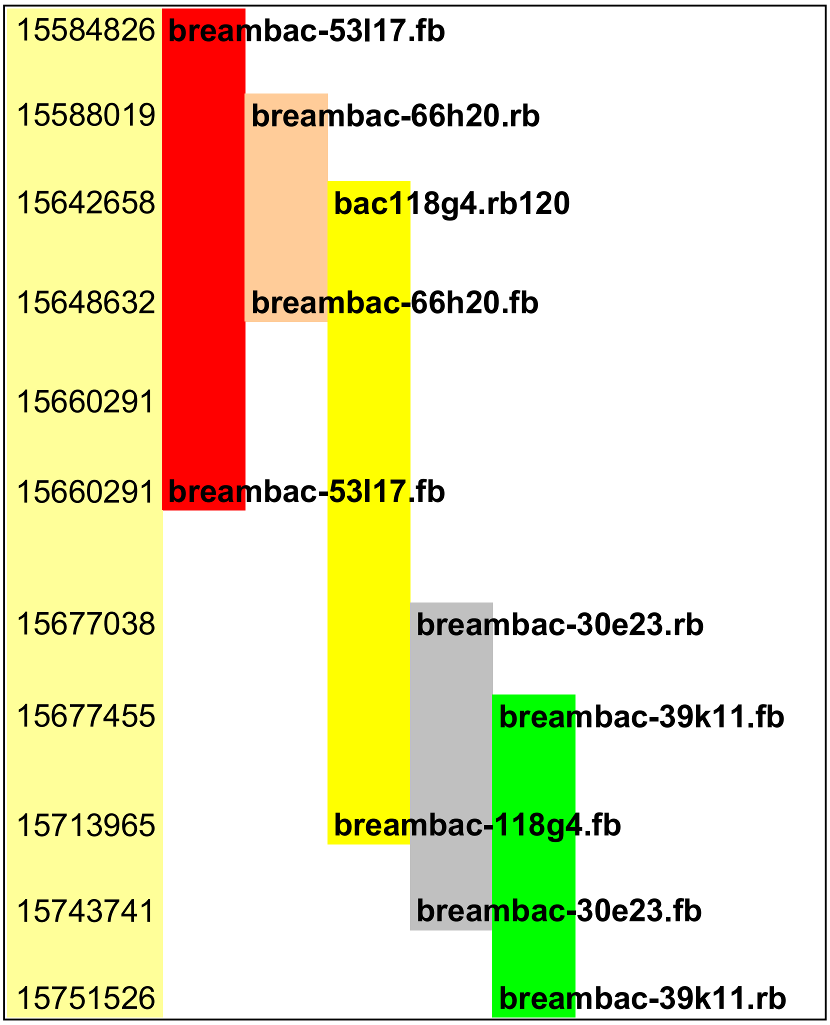

Supplemental file 3
Alignement of candidate BAC clones isolated around the candidate region for QTL (Did12 breambac118g4) affecting growth-related traits as well as sex reversal onto the genome of Stickleback.
