# Supplementary material for: Quantitative Trait Loci Involved in Sex Determination and Body Growth in the Gilthead Sea Bream (Sparus aurata L.) through Targeted Genome Scan
Source: PLoS One. 2011 Jan 31;6(1):e16599. doi: 10.1371/journal.pone.0016599 (PMC3031595; doi:10.1371/journal.pone.0016599)
Supplement: Figure S4 — Alignment of marker sequence Did12 and BAC sequences of Sparus aurata breambac-118g4. In gray are shown the corresponding primer sequences mapped 1.8 cM away from each other according to linkage mapping analysis (Fig. 1). (PDF) [file pone.0016599.s004.pdf]

```
DId12                     ACAAACTGCGTGAAGCTTAGCAACTTTCTTCTTCTCATCCACCTTGTNGT
breambac-118g4            ------------------AGCAACTTTCTTCTTCTCATCCACCTTGTCGT

DId12                     TTGCACATCAAAGCCATATAGATAAGGCATTTACACACACACACACGCAC
breambac-118g4            TTGCACATCAAAGCCATATAGATAAGGCATTTACACACACACACACGCAC

DId12                     ACACACACACCAGCTTGTTCATCACAAACACACGCTCTTAACTCATCAAA
breambac-118g4            ACACACACACCAGCTTGTTCATCACAAACACACGCTCTTAACTCATCAAA

DId12                     CTCTAACCACAGCTGCTGCAGTAAAACCACGTTGACTAACGAGAAAAAAC
breambac-118g4            CTCTAACCACAGCTGCTGCAGTAAAACCACGTTGACTAACGAGAAAAAAC

DId12                     CAGCGTGCCGCTTACAGACTCCCCACTCCTACTGTACCTTCAAAACTTAA
breambac-118g4            CAGCGTGCCGCTTACAGACTCCCCACTCCTACTGTACCTTCAAAACTTAA

DId12                     CCATAACACACACACATTTGGACGTAAACACACACACACACACATGCTCA
breambac-118g4            CCATAACACACACACATTTGGACGTAAACACACACACACACACATGCTCA

DId12                     CACACACCATTACTCTTTCTTTCTCTCTATCTACTACGTTTAATTCCCTT
breambac-118g4            CACACACCATTACTCTTTCTTTCTCTCTATCTACTACGTTTAATTCCCTT

DId12                     TGGAAAAGACAGGTGCAAACGTTGATTGAGCCTGAGCCAGATTTAGCCCC
breambac-118g4            TGGAAAAGACAGGTGCAAACGTTGATTGAGCCTGAGCCAGATTTAGCCCC

DId12                     AGCTCTCAGTGTGCGTCCAGCTGTACTCTCCAACCCACTGAGA-------
breambac-118g4            AGCTCTCAGTGTGCGTCCAGCTGTACTCTCCAACCCACTGAGATCGATGT
```

Supplement Figure 4

Alignement of marker sequence DId12 and BAC sequences of seabream BAC 118g4.

In grey the corresponding primer sequences mapped 1.8 cM away from each other on the genetic linkage map.
